# Glass-ceramic optical fiber containing Ba_2_TiSi_2_O_8_ nanocrystals for frequency conversion of lasers

**DOI:** 10.1038/srep44456

**Published:** 2017-03-30

**Authors:** Zaijin Fang, Xusheng Xiao, Xin Wang, Zhijun Ma, Elfed Lewis, Gerald Farrell, Pengfei Wang, Jing Ren, Haitao Guo, Jianrong Qiu

**Affiliations:** 1 Key Lab of In-fiber Integrated Optics, Ministry Education of China, Harbin Engineering University, Harbin 150001, China; 2 State Key Laboratory of Luminescent Materials and Devices, South China University of Technology, Guangzhou 510640, China; 3 State Key Laboratory of Transient Optics and Photonics, Xi’an Institute of Optics and Precision Mechanics, Chinese Academy of Sciences, Xi’an 710119, China; 4 Graduate School of Chinese Academy of Sciences, Beijing 100049, China; 5 Optical Fibre Sensors Research Centre, Department of Electronic and Computer Engineering, University of Limerick, Limerick, Ireland; 6 Photonic Research Centre, Dublin Institute of Technology, Kevin Street, Dublin 8, Ireland; 7 College of Optical Science and Engineering, State Key Laboratory of Modern Optical Instrumentation, Zhejiang University, Hangzhou 310027, China

## Abstract

A glass-ceramic optical fiber containing Ba_2_TiSi_2_O_8_ nanocrystals fabricated using a novel combination of the melt-in-tube method and successive heat treatment is reported for the first time. For the melt-in-tube method, fibers act as a precursor at the drawing temperature for which the cladding glass is softened while the core glass is melted. It is demonstrated experimentally that following heat treatment, Ba_2_TiSi_2_O_8_ nanocrystals with diameters below 10 nm are evenly distributed throughout the fiber core. Comparing to the conventional rod-in-tube method, the melt-in-tube method is superior in terms of controllability of crystallization to allow for the fabrication of low loss glass-ceramic fibers. When irradiated using a 1030 nm femtosecond laser, an enhanced green emission at a wavelength of 515 nm is observed in the glass-ceramic fiber, which demonstrates second harmonic generation of a laser action in the fabricated glass-ceramic fibers. Therefore, this new glass-ceramic fiber not only provides a highly promising development for frequency conversion of lasers in all optical fiber based networks, but the melt-in-tube fabrication method also offers excellent opportunities for fabricating a wide range of novel glass-ceramic optical fibers for multiple future applications including fiber telecommunications and lasers.

Since optical fibers were introduced in 1960s, they have subsequently attracted much attention for a range of applications such as fiber amplifiers and fiber lasers^1^,^2^,^3^. The material of choice for optical fibers has expanded from silica glass only to now including compound glasses in order to satisfy the growing demands for new optical devices working in different wavelength ranges. Glass-ceramic (GC) is a two-phase composite in which crystals are homogeneously distributed throughout the glass matrix. It exhibits high transmittance as well as strong crystal field if active ions are successfully doped in the crystalline lattices. These features make GC a suitable gain material for optical fibers to achieve enhanced emission, broadband emission and other desirable features^4^,^5^,^6^,^7^,^8^,^9^,^10^. Previously, nonlinear GC has attracted much attention for frequency conversion of pulse lasers^11^,^12^,^13^,^14^,^15^,^16^,^17^,^18^. Fresnoite (Ba_2_TiSi_2_O_8_) crystal has been extensively studied because it exhibits good dielectric and nonlinear optical properties. GCs containing Ba_2_TiSi_2_O_8_ crystals possess significant second-order optical nonlinearities, comparable to that of LiNbO_3_ single crystals, which has been demonstrated by Takahashi *et al*.^19^. GC optical fiber containing Ba_2_TiSi_2_O_8_ crystals is also a promising material for second harmonic generation (SHG) of lasers without a dependence on the incident wavelength of the host fiber-network. To date, no nonlinear GC optical fiber with excellent performance has been reported, which has been attributed to the difficulty in fabricating GC optical fibers.

Very recently, a concerted effort has been directed towards fabricating GC fibers. Rare-earth doped oxyfluoride GC fibers have been investigated because of their low-photon-energy environment^20^,^21^,^22^. Additionally, Ni^2+^- doped GC fiber^23^, Cr^4+^-doped forsterite GC fiber^24^, and other GC fiber have also been reported^25^. The fabrication techniques of these GC fibers were mostly based on a rod-in-tube method, in which the fiber core and clad glass are similar in composition, and the fiber is drawn near the softening temperature of fiber core glass. Generally, the softening temperature of glass is higher than the peak crystallization temperature. The crystals in the fiber core glass grow rapidly because the crystallization barrier of glass component is low at the softening temperature. This crystallization process is uncontrollable, and the sizes of the resulting crystals are so large that the scattering of particles with different refractive indices in the glass matrix can become significant. Thus, the transmission loss of the fiber core glass in this case is high and therefore the rod-in-tube method may not suitable for fabricating GC fibers. In order to improve the process, a novel method is proposed in this investigation for the fabrication of Ba_2_TiSi_2_O_8_ GC optical fibers. The proposed method is an analog to the conventional rod-in-tube process, and it is called the melt-in-tube method. The novel fibers fabricated using this method were characterized using electro-probe micro-analyzer (EPMA) imaging, Micro-Raman spectrum analysis and high-resolution transmission electron microscopy (HRTEM) imaging techniques. Using these techniques the element distribution and microstructures were accurately identified. The SHG of the laser action in the GC optical fiber was demonstrated by measuring the emission spectra when irradiated using a 1030 nm femtosecond laser.

## Experiments

The core material for the precursor fiber was a silicate multi-component glass with the compositions (mol%): 45SiO_2_-5Al_2_O_3_-35BaO-15TiO_2_, which was chosen as a candidate precursor glass for the precipitation of Ba_2_TiSi_2_O_8_ nanocrystal^17^. A 200 g reagent grade stoichiometric mixture of SiO_2_, Al_2_O_3_, BaCO_3_ and TiO_2_ was mixed thoroughly in an agate mortar and melted in a Pt-Rh crucible at 1650 °C for 2 h. The glasses were fabricated by pouring the melt into a preheated mould, the glass was annealed at 600 °C for 3 h to relieve inner stress. The density of the fiber core glass was measured to be 3.9 g/cm^3^, the refractive index was 1.68 at 632 nm and the thermal expansion coefficient was 3.6 × 10^−6^/K. The glass block was cut into two parts, which were cold worked in a lathe into two cylindrical rods with a diameters of 15.0 and 2.0 mm. The surfaces of the rods were polished and etched by hydrofluoric acid in order to remove the contaminated surface layer.

Before the fabrication of the GC optical fiber, the thicker glass rod (diameter of 15.0 mm) was suspended in a resistance furnace and drawn into fibers for determining the ability of the material to withstand crystallization. The drawing temperature was near the glass softening temperature (~960 °C). Following this, the GC fibers were fabricated by the melt-in-tube method. Firstly, the thinner glass rod (diameter of 2.0 mm) was inserted into a high-purity (99.999%) silica glass cylindrical tube with inner diameter of 2.1 mm and external diameter of 30.0 mm. The bottom of tube was sealed to form a preform. The preform was suspended in a graphite furnace and the temperature was rapidly raised to about 1830 °C. By quickly drawing (at a rate of ~15 m/min), the precursor optical fibers were prepared. Finally, the precursor optical fibers were heat treated at about 850 °C to obtain the GC optical fiber.

Differential thermal analysis (DTA) experiments were performed in an STA449C Jupiter (Netzsch, Bavaria, Germany) oven in an argon atmosphere and a heating rate of 10 K/min in the range from 25 to 1100 °C. The amorphous state and crystalline phase in the glasses were identified using X-ray diffraction (XRD) on a D8 advance X-ray diffractometer (Bruker, Faellanden, Switzerland) with Cu/Kα (λ = 0.1541 nm) irradiation. The XRD patterns of the samples were collected in the range of 10° <2θ <70°. For determining the element distribution of the precursor fiber, the fiber samples were measured using an EPMA system (EPMA-1600, Shimadzu, Kyoto, Japan). Micro-Raman spectra and mapping images were recorded using a Raman spectrometer (Renishaw inVia, London, UK) excited by a 785 nm laser. The morphology and size distribution of the nanocrystals in GC optical fibers were measured using a HRTEM instrument (Tecnai G2, FEI, Amsterdam, Netherlands). A commercial femtosecond Yb-fiber laser system (FLCPA-02USCT11, Calmar Laser, Palo Alto, California, USA) emitting 370 fs, 1030 nm laser pulses at a repetition rate of 500 kHz was employed to induce SHG in the optical fibers. The emission spectra of the optical fibers during the femtosecond laser irradiation were recorded using a spectrometer (HR4000, Ocean Optics, Winter Park, Florida, USA). All measurements were performed at room temperature.

## Results and Discussion

**Figure 1:**
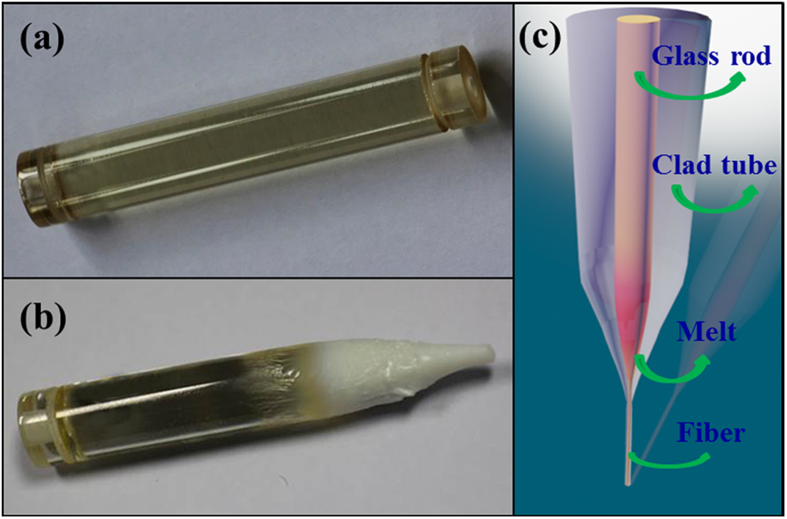
Images of the glass preform (**a**) before and (**b**) after the drawing process, (**c**) schematic diagram of the melt-in-tube fiber drawing technique.

**Figure 2:**
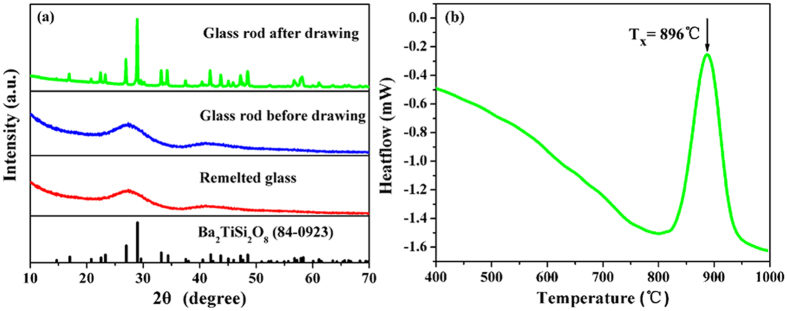
(**a**) XRD patterns of the glass rods before and after the drawing process and remelted glass. (**b**) DTA curve of the fiber core glass.

Figure 1(a) and (b) show images of the thicker glass rod before and after the drawing process at about 960 °C. Before drawing, the glass rod is transparent across its entire length. The glass rod exhibits an amorphous state, which is confirmed by the XRD distribution as shown in Fig. 2(a). After the drawing process, significant crystallization is in evidence at the bottom of the glass rod as shown in Fig. 1(b), which was located in the high-temperature region of resistance furnace during the drawing process. The crystals can be identified as Ba_2_TiSi_2_O_8_ (JCPDS Card No. 84-0923) by the XRD pattern as shown in Fig. 2(a). The diffraction peaks of Ba_2_TiSi_2_O_8_ crystals are all narrow, which indicates that the sizes of the crystals are large. Scherrer’s equation was used to estimate the size of the crystals. The strongest diffraction peak, around 2θ = 28.9°, was selected for the calculation, and the average diameter of the Ba_2_TiSi_2_O_8_ crystals was calculated to be approximately 61.2 nm. These relatively large particles significantly impair optical transmission in fibers due to increased scattering, and the opaque appearance of the glass rod shown in Fig. 1(b) can be explained by the thermal analysis result.

According to the DTA curve presented in Fig. 2(b), the onset and peak crystallization temperatures (T_x_) of the core glass occur at about 800 °C and 900 °C, respectively, which are lower than the drawing temperature of the fiber (~960 °C). At the fiber drawing temperature, the crystals grow rapidly. Thus, it is difficult to avoid uncontrollable crystallization of glass during the fiber drawing process. Similarly, the drawing temperature of the fiber by the rod-in-tube method is also near the softening temperature of the fiber core glass (~960 °C), which in turn results in the increase of transmission loss caused by the uncontrollable crystallization. Consequently, the rod-in-tube method is not suitable for fabricating this Ba_2_TiSi_2_O_8_ GC fiber.

However, when the crystallized glass rod was remelted at 1650 °C the glass became transparent again after quenching, i.e., the crystals were dissolved during the secondary melting process, and the rod glass became amorphous again as confirmed by the XRD pattern as shown in Fig. 2(a). Based on this fact, the GC optical fibers were prepared using the melt-in-tube method followed by a subsequent thermal treatment, and the harmful consequences of crystallization can be completely avoided because all precipitated crystals are dissolved during the melt-in-tube process. The schematic diagram of the melt-in-tube technique is shown in Fig. 1(c). The fiber clad glass was softened while the fiber core glass melted at the fiber drawing temperature (~1830 °C).

**Figure 3:**
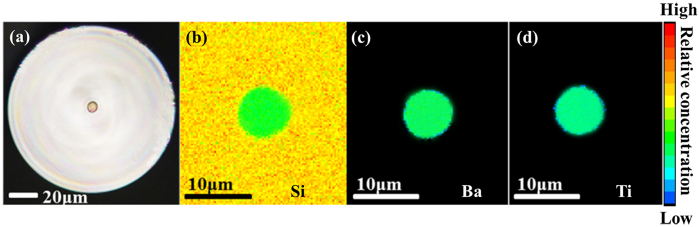
(**a**) Cross section image and (**b**–**d**) EPMA images of the precursor fiber.

In order to determine the element distribution in the precursor fiber, the optical and EPMA images of fiber cross section were captured and are shown in Fig. 3. It is observed from Fig. 3(a) that, the fiber and fiber core both exhibit excellent circular concentricity and uniformity. No cracking have been observed in both fiber core and clad. The diameter of fiber and fiber core are about 125.1 and 8.5 μm respectively, which is approximately equal to the original proportion of the ratio of core/cladding in the preform. The interface between the fiber core and clad is clear and the waveguide structure of fiber is well maintained after the drawing process. These results can be explained by EPMA mapping images as shown in Fig. 3(b–d). Ba and Ti are the only elements distributed in the fiber core, Si exhibits a step distribution due to its different concentration values in the fiber core and cladding. This element distribution is consistent with the original compositions of the core and cladding glasses. The boundaries of element distributions are all circular in appearance, and the diameters of the boundaries are well matched to that of fiber core (8.5 μm). These results indicate that there was no obvious element diffusion between fiber core and cladding during the drawing process, which can be attributed to the fast fiber drawing speed used.

**Figure 4:**
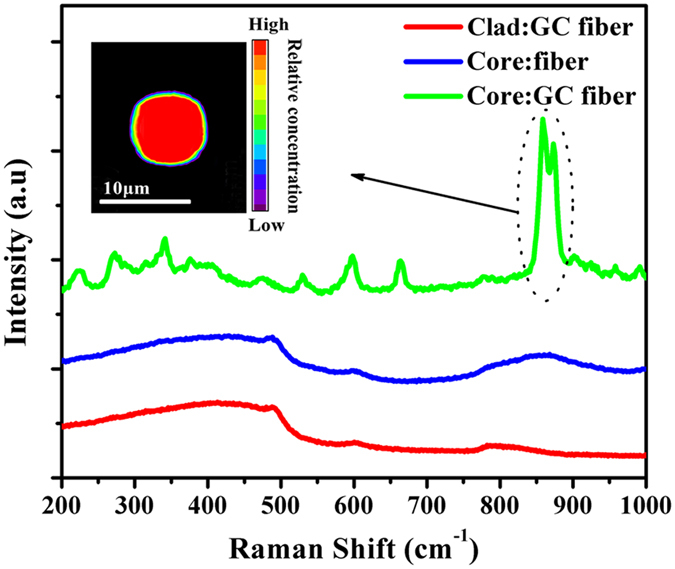
Micro-Raman spectra of the precursor and GC fibers at different regions, the inset is the mapping pattern at the cross section of GC fiber.

For the characterization of microstructure in fibers, the Micro-Raman spectra of the fiber’s cross section were measured and are shown in Fig. 4. The focus diameter and the power of the probe laser were 1 μm and 25 mW, respectively. From Fig. 4, one can clearly see that the Raman spectrum of the precursor fiber core consists of several broad bands over the spectrum. The broad band located around 500 cm^−1^ corresponds to the Si-O-Si bending vibration modes of the [SiO_4_] unit^26^,^27^,^28^,^29^. The band located around 600 cm^−1^ is associated with the Si-O-Si symmetric stretching vibration of [SiO_4_] unit^30^,^31^,^32^. The band ranging from 800 to 1000 cm^−1^ can be associated with the stretching mode of the short Ti-O* bond (O* denotes an apical oxygen), the Ti-O bonds (O-denotes a non-bridging oxygen)^33^. No sharp crystal peak was observed in the Raman spectrum of precursor fiber core indicating that the fiber core is in an amorphous state after the fiber drawing process. Consequently, the melt-in-tube technique can be used to avoid the uncontrollable crystallization during fiber drawing process.

According to the DTA curve of fiber core glass, the precursor fibers were heat treated at 850 °C for 5 hours to obtain GC fibers. The crystals appear in fiber and are detected by Raman spectra as shown in Fig. 4. Very clear spectroscopic changes can be observed in the Raman spectrum of GC fiber core compared to that of precursor fiber core. For the Raman spectra of the GC fiber core, the band ranging from 800 to 1000 cm^−1^ becomes narrow and a strong sharp peak is observed at about 860 cm^−1^. Additionally, the band around 300 cm^−1^ in the Raman spectrum splits into several peaks at 222, 273, 343, and 375 cm^−1^ which are attributable to the translational and bending modes of the Si_2_O_7_ and TiO_5_ groups^34^. Three peaks at 531, 598 and 663 cm^−1^ are also observed in the Raman spectrum of the GC fiber core, which are assigned to the ν (TiO_4_) and ν_s_ (Si-O-Si) modes^35^. All of the sharp peaks are in good agreement with the previously observed Raman spectrum of Ba_2_TiSi_2_O_8_ crystals^33^. These results indicate that Ba_2_TiSi_2_O_8_ crystals have precipitated in the fiber as a result of heat treatment at 850 °C for 5 h. The Raman spectrum of the GC fiber cladding is also presented in Fig. 4, which shows several bands corresponding to the characteristic Raman spectrum of silica glass^36^. The band around 500, 600 and 800 cm^−1^ is associated with the Si-O-Si bending vibration modes of the [SiO_4_] unit, Si-O-Si symmetric stretching vibration of the [SiO_4_] unit and asymmetric stretching vibration of Si-O-Si network. No clear crystal peak is observed in the Raman spectrum of the GC fiber cladding. These results indicate that the crystallization in GC fiber can therefore be confined to the fiber core.

Furthermore, the Raman mapping pattern around the core of the GC fiber was measured to provide a detailed investigation in the definite spatial distribution of the precipitated Ba_2_TiSi_2_O_8_ crystals, which is shown in the inset of Fig. 4. The intensity of the most intense characteristic Raman peak at 860 cm^−1^ relative to the baseline was used as the detecting signal, which represents the distribution of the Ba_2_TiSi_2_O_8_ crystals. It can be observed that the distribution of the Ba_2_TiSi_2_O_8_ crystals are confined to a circle with diameter of about 8.1 μm, which corresponds well to the extent of the fiber core (8.3 μm). It has also been observed that the Ba_2_TiSi_2_O_8_ crystals are evenly distributed within the fiber core, indicating that the precipitation process in the fiber core is uniform and not due to any interfacial crystallization.

**Figure 5:**
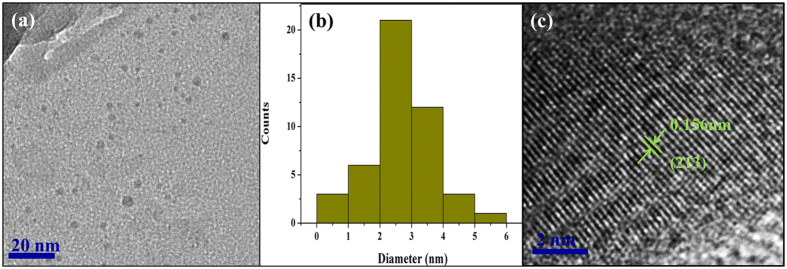
(**a**) TEM image of GC fibers with a heating treatment at 850 °C for 5 hours. (**b**) The size distribution of crystals in GC fibers corresponding to the TEM image (**a**). (**c**) HR-TEM image of the GC fiber.

For the GC fibers, high transmittance is important as well as maintaining controllable crystallization. Considering the likely application areas of the GC fiber, the size of the crystals must be as small as possible to reduce the transmission loss caused by light scattering due to the presence of particles with a different refractive index from the host glass matrix. To observe the morphology and size distribution of crystals in the GC fiber, the GC fiber samples were ground into powders and measured using TEM and HRTEM imaging. Figure 5(a) shows that the particles are uniformly dispersed in the glass matrix. Figure 5(b) shows that the diameter of crystals lies from 1.0 to 6.0 nm, with the majority being between 2 to 4 nm. Tick *et al*.^37^ proposed that the scattering losses are minimal when the sizes of the nanocrystals in the GC are smaller than the 1/20 of transmission wavelength of light (therefore corresponding to a diameter of the order of 10 nm). Therefore, the transmission loss caused by light scattering of the nanoparticles in this GC fiber is expected to be rather weak. The light transmission loss of the glass ceramic fibers fabricated using a heating treatment at 850 °C for 5 hours has been measured using the cutback method. The value of transmission loss was measured as 0.081 dB/cm at 532 nm. The HRTEM image of one single crystal from Fig. 5(a) is shown in Fig. 5(c). The crystal lattice fringes are clear, which are different from that of the amorphous glass matrix. The interval of the crystal lattice fringes can be measured directly, and its value is about 0.156 nm, which corresponds to the (213) crystal facet of Ba_2_TiSi_2_O_8_. This result also proves the precipitation of Ba_2_TiSi_2_O_8_ crystals in the GC fibers.

**Figure 6:**
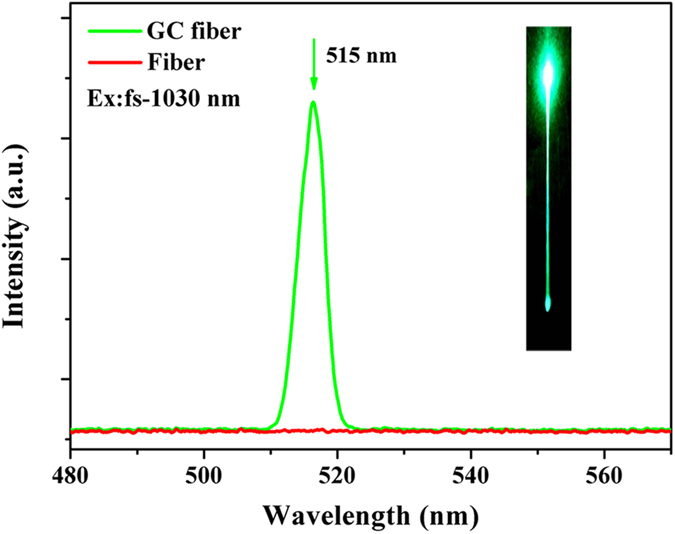
The emission spectra of the precursor and GC fiber heat treated at 850 °C for 5 hours. The inset is the image of the GC fiber irradiated using the 1030 nm femtosecond laser.

To demonstrate the frequency conversion of laser in fibers, the emission spectra of the precursor and GC fiber were measured and are shown in Fig. 6. When irradiated using a 1030 nm femtosecond laser with power of 100 mW, no clear emission was observed in the spectrum of precursor fiber. For the GC fiber, the fiber turns green along its entire length as shown in the inset of Fig. 6. An intense visible emission peak was observed in the spectrum of GC fiber, the center of emission peak being located at a wavelength of 515 nm. This enhanced emission indicates that the GC fiber containing Ba_2_TiSi_2_O_8_ nanocrystals can be used for the frequency conversion of the femtosecond laser.

In order to investigate the mechanism of the frequency conversion in the Ba_2_TiSi_2_O_8_ GC fiber, the emission spectrum dependence on the irradiation power was measured. Generally, the energy of one infrared pumping event is not sufficient to achieve a visible emission in a single-photon process. The visible emission from Ba_2_TiSi_2_O_8_ GC fiber is therefore likely to originate from at least a two-photon excitation.

**Figure 7:**
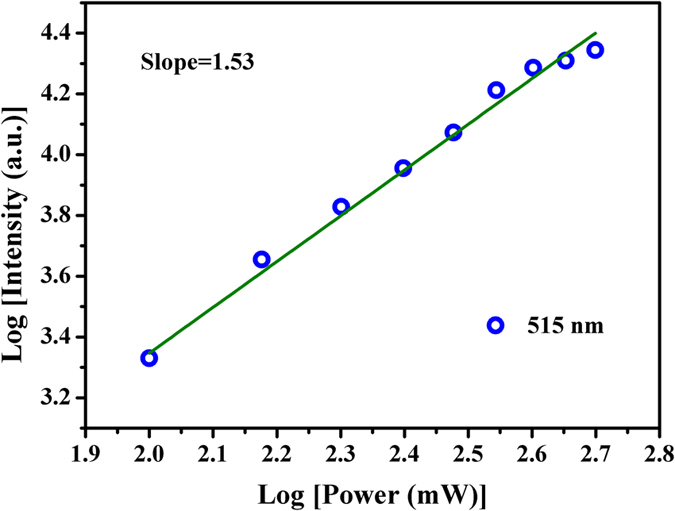
Double-logarithmic plot of the irradiation power dependency on the 515 nm emission intensity from the Ba_2_TiSi_2_O_8_ GC fiber.

It is well known that the relationship between the emission intensity and the irradiation power can be described by Equation (1):


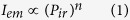


where I_em_ is the intensity of the visible emission, P_ir_ is the irradiation power of the femtosecond laser, n is the absorbed photon numbers per visible emitted. The values of n can be obtained from the fitted slope of the plot of log (I_em_) versus log (I_ir_). Based on these, the double-logarithmic plot of emission intensity dependence on irradiation power was measured and is shown in Fig. 7. The logarithm of the 515 nm emission intensity increases linearly with the logarithm of irradiation power, the fitted linear slope is found to be 1.53. As shown in Fig. 6, the center of emission peak is at the half of irradiation wavelength. Therefore, the frequency conversion of lasing in GC fiber originates from a two-photon process, which is due to the SHG of femtosecond laser output when the Ba_2_TiSi_2_O_8_ nanocrystals in the GC fiber core are irradiated.

Additionally, the frequency conversion efficiency (*η*) can be estimated according to the Equation (2):


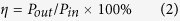


where *P*_*out*_ and *P*_*in*_ represent the optical power produced by the output and input laser, respectively. The estimated *η* of the GC fiber heat treated at 850 °C for 5 hours is about 1.2% when irradiated by 900 mW femtosecond laser. Work is ongoing to increase the frequency conversion efficiency of GC fiber, for example through changes to the compositions of the fiber core and cladding glasses, improvements in the coupling between the irradiation laser and the glass ceramic fiber and finally through the refinement of the fiber treatment techniques, such as the heat treatment process.

## Conclusion

In this work, the GC fibers containing Ba_2_TiSi_2_O_8_ nanocrystals were prepared using the melt-in-tube method and successive heat treatment. The precursor fibers were fabricated without any appearance of element inter-diffusion or crystallization. After heat treatment, the GC fibers were fabricated with the result that Ba_2_TiSi_2_O_8_ nanocrystals were distributed evenly throughout the fiber core, the diameter of nanocrystals being between 1.0 and 6.0 nm which is sufficiently small to ensure low transmission loss due to scattering. Comparing to the conventional rod-in-tube method, the melt-in-tube method is a secondary melting process and allows controllable crystallization for fabricating GC fibers. When irradiated using a 1030 nm femtosecond laser, an enhanced green emission (515 nm) was observed for the first time in the glass ceramic fiber compared to that of precursor fiber, which is evidence of second harmonic laser generation in the GC fiber. Therefore, this GC fiber represents a promising matrix for frequency conversion of lasers in modern optical fiber-networks. The melt-in-tube fabrication method also represents an entirely new process for fabricating GC fibers with low loss properties that are not achievable using the more conventional methods referred to earlier in this paper.

## Additional Information

**How to cite this article:** Fang, Z. *et al*. Glass-ceramic optical fiber containing Ba_2_TiSi_2_O_8_ nanocrystals for frequency conversion of lasers. *Sci. Rep.* **7**, 44456; doi: 10.1038/srep44456 (2017).

**Publisher's note:** Springer Nature remains neutral with regard to jurisdictional claims in published maps and institutional affiliations.
